# Using Support Vector Machine on EEG for Advertisement Impact Assessment

**DOI:** 10.3389/fnins.2018.00076

**Published:** 2018-03-12

**Authors:** Zhen Wei, Chao Wu, Xiaoyi Wang, Akara Supratak, Pan Wang, Yike Guo

**Affiliations:** ^1^Data Science Institute, Imperial College, London, United Kingdom; ^2^School of Public Affairs, Zhejiang University, Hangzhou, China; ^3^School of Management, Zhejiang University, Hangzhou, China

**Keywords:** EEG, SVM, advertisement impact assessment, neuromarketing, machine learning

## Abstract

The advertising industry depends on an effective assessment of the impact of advertising as a key performance metric for their products. However, current assessment methods have relied on either indirect inference from observing changes in consumer behavior after the launch of an advertising campaign, which has long cycle times and requires an ad campaign to have already have been launched (often meaning costs having been sunk). Or through surveys or focus groups, which have a potential for experimental biases, peer pressure, and other psychological and sociological phenomena that can reduce the effectiveness of the study. In this paper, we investigate a new approach to assess the impact of advertisement by utilizing low-cost EEG headbands to record and assess the measurable impact of advertising on the brain. Our evaluation shows the desired performance of our method based on user experiment with 30 recruited subjects after watching 220 different advertisements. We believe the proposed SVM method can be further developed to a general and scalable methodology that can enable advertising agencies to assess impact rapidly, quantitatively, and without bias.

## 1. Introduction

Advertising plays a critical role in marketing. Every year, companies allocate a significant proportion of marketing budget to attempt to quantify the impact of their advertising, particularly video advertising on TV and on the web which receives wide viewership (Brady, 2014; Bradley, 2015). However, current methodologies, including both direct observation (questionnaires and focus groups prior to starting of the advertising campaign), and indirect (trends in sales or consumer interest during and after a campaign), tend to have practical or experimental challenges that reduce the effectiveness of the assessment (Goldberg, 1990; Ducoffe, 1996; Elliott and Speck, 1998; Lewis and Reiley, 2009; Ostrovsky and Schwarz, 2011).

Direct observation methods include questionnaires, and focus groups, where yet to be released advertisements (or multiple versions of an advertisement) are shown to a select group of viewers selected to be representative of the advertisement's intended audience. The viewers answer questions and provide feedback during a survey or engage in discussion with the organizers; then the results or discussion are analyzed by the advertising team to try to assess how well the advertisement fulfils the criteria of their campaign (Gaines et al., 2007).

However, direct approach methods are subject to the same kinds of challenges as other experimental psychology approaches: experimental biases are introduced due to the experimental environment (typically an office room with multiple participants) being different to how a viewer would normally view the advert (often in the comfort of one's own home), leading to a different state of mind of the viewer. There is a tendency for respondents to feel obliged to give more favorable reviews under experimental conditions of being under observation than they would typically do. The way the experiment is carried out in groups can also result in peer-pressure and group-dynamics altering the responses of individuals, leading to participants reporting attitudes or preferences that may not truly represent their own when they are in private (De Pelsmacker et al., 2002; Maison et al., 2004; Shen and Li, 2009). The analysis of survey feedback or discussion too can introduce experimenter-bias where the opinions of the experimenters impact the evaluation of the results; and the size and cost of conducting the study and analyzing the results is often cost-prohibitive past certain scales (and when considering TV advertising for national release, the size of these focus studies are often a tiny proportion of eventual viewership, resulting in uncertainty of the statistical relevance of the results).

Indirect observation methods of assessing advertising impact involve inferring advertising impact based on the result of an advertising campaign (Sharma et al., 2011). These methods avoid the inaccuracies imposed by artificial experimental conditions of direct observation methods but suffer from their own challenges. By looking only at the results and effects of the advertisement campaign, and because it is only possible to look at aggregated effects (such as the impact on sales or customer interest in a product), it is often difficult to identify if, why, and how a particular aspect of the advertisement causes an impact. Furthermore due to the life-cycle of an advertising campaign, it is only possible to infer impact during or after the launch of an advertising campaign, meaning in many cases, much or all of the cost of the campaign having been sunk, limiting adaptability in the event of lackluster response to the campaign (Kanetkar et al., 1992; Grewal et al., 1998; Sundar and Kalyanaraman, 2004).

In recent decades, research in neuroscience has brought new understanding and tools that can change how the impact of advertising can be assessed and has formed the new field of neuromarketing, in which recent neuroscience and experimental psychology tools and understanding are being applied to marketing. One key hypothesis in neuromarketing is that a consumers decisions can be driven more by emotion than by a careful comparison of product benefits or differentiators. Therefore, measuring an advertisement's emotional impact on an individual could correlate well with the impact of the advertisement.

In neuromarketing-based advertisement impact assessment, biometrics are gathered from individuals participation in the study, and these biometrics are used to assess impact, rather than voluntarily self-reported information from surveys or discussion. The recorded data includes biometrics such as eye-tracking, facial coding, Galvanic skin response and electrodermal activity, and EEG. EEG is a noninvasive electrophysiological recording of brain activity, using electrodes placed along the scalp. EEG has multiple advantages over other methods of measuring brain activity in that it has a high temporal resolution, is non-invasive, quick to instrument and tolerant to subject movement, and low cost with the use of single electrode equipment. In 2010, Murugappan found in a study of human-computer interaction, a correlation between a user's emotion and EEG, providing useful information in understanding a user's reaction to advertisements (Murugappan et al., 2010). Therefore, an increasing amount of research into neuromarketing has turned to EEG as a key sensor in measuring emotion. Lucchiari and Pravettoni observed that EEG signals with a frequency of 16–31 Hz (i.e., Beta wave) could be modulated by the experience of pleasure when a consumer was presented with a favorite brand (Lucchiari and Pravettoni, 2012). In 2016, Wang, Chang and Chuang found that a narratives structure in video commercials induced higher EEG signals with a frequency band of 4–7 Hz (i.e., higher Theta) power of the left frontal region resulting in higher preference for branded products (Wang et al., 2016).

In psychology research, a person's emotion can be quantified through self-reported measures such as liking (valence) and excitability (awaken) (Poels and Dewitte, 2006; Smit et al., 2006). Questionnaires can be used to gather this type of information. According to the AIDA model, four quantified metrics are used to characterize the experience for a consumer watching an advertisement: attention, interest, desire, and action (Strong, 1925).

In the literature, Support Vector Machine has been widely used on EEG data; research on EEG based emotion recognition using frequency domain features and Support Vector Machine (SVM) was done by Wang et al. (2011)Research on EEG-based emotion recognition in music listening using Support Vector Machine(SVM) was done by Lin et al. (2009) Though other regression in binary results can also be used to build this model, in the literature, Support Vector Machine is the most widely used method in this field. Also, Support Vector Machine is suitable because of the sparse dataset the experiment uses. The technique used will build a prediction model based on several different brainwaves, which include frequency band less than 4Hz (i.e. Delta), frequency band between 4 and 7 Hz (i.e., Theta), frequency band 8–15 Hz (i.e., Alpha), frequency band 16–31 Hz (i.e., Beta) and frequency bigger than 32Hz (i.e., Gamma).

In this paper, we test the hypothesis that it is possible to use low-cost EEG equipment to collect brainwaves of subjects viewing advertisement, and to apply the latest methods from neuromarketing, and machine learning as a more accurate method of assessing advertisement impact and the likelihood of a person purchasing the advertised product than the current state-of-the-art.

## 2. Methodology

This section of the paper will describe the method used to collect data from a single-electrode wearable EEG device, self-reported measures for the impact of an advertisement, and train a predictive model against the data using SVM (Poels and Dewitte, 2006; Smit et al., 2006; Chen et al., 2014).

### 2.1. Data collection

In the experiment, thirty right-handed male participants aged 20–35 from the University participated in this experiment as paid volunteers. Thirty participants were in the experiment, each of them is given 4–5 advertisements, to create a sample size of 450, big enough to carry out statistical analysis. The experiment was carried in China, and the participants are bi-lingual in Chinese and English speaker. The participants had normal or corrected vision without any histories of neurological/mental diseases. The purpose of choosing bi-lingual participants is to avoid language-barrier caused by the advertisement's language content being in Chinese or English. We selected male participants to avoid biases from gender-specificity of the advertised products. Many products are specifically targeted at a particular gender, for example, male clothing and female clothing are targeted differently; as are hair and cosmetic products. We constructed a database of 220 TV advertisements from four gender-neutral or male-targeted products: cars (55 ads), digital products (55 ads), clothing (55 ads) and food (55 ads), which were randomly selected from Youku.com (one of the largest online media websites). Each advertisement was 15–20 s in length. The video resolution and audio volume of each video were normalised to the same level using professional video and audio editing software. In each test, 4–5 advertisements were randomly selected from the 220 TV advertisements database. The shortcomings and future research because of this design will be discussed in section 4.

Before the experiment, the volunteers received detailed instructions on all the tasks they would perform. Each participant was fitted with a single-electrode EEG headset by an experimenter, was seated comfortably in a lab room at 1.20 meters from a 19-inch PC monitor, and shown five advertisements randomly selected from every genre for a total of 20 advertisements. An E-prime system was used to control the presentation of the stimuli.

The experiment consisted of 4 blocks, each containing 20 trials. During every trial, the volunteers were presented with the advertising for about 15–20 s. The advertisement was followed by an evaluation questionnaire, including the willingness of the participant to purchase the advertised product (yes or no), and liking the advertisement (7-point Like scale).

Each volunteer performed two practice trials before the start of the formal experiment. The frontal EEG was recorded with the single-channel dry electrode-device and system (NeuroCAR1.0, Neuromanagement Lab, Zhejiang University, China). The integrated chip of the device was the ThinkGear (NeuroSky, Wuxi, China). The sampling rate of the device for gathering EEG signals was 512 HZ and the data saved into a computer. Four trials data were rejected due to voltage abnormality, and in total, 450 sets of brainwave data were used in our study.

### 2.2. Questionnaire collection

The participants were asked to fill out a questionnaire with 22 questions asking about different aspects (objective and subjective) of the advertisement, or their experience. The questionnaire is listed in Table 1. The questionnaires are chosen to record different aspects of the advertising can impact the viewer's response to the advertising, which may indicate the likelihood of the viewer wanting to purchase the product. The aspects are chosen from several studies in the literature that each focus on one of two aspects of an advertisement's impact. M. Vaismoradi, Kimberly, and Klaus show that the audio and visual fidelity of the advertisement (Y2 and Y15 of the questionnaire) had an impact (Vaismoradi et al., 2013). The content impact are covered by questions Y1, Y5, and Y14. The brand quality is covered in (Y6, Y7, and Y8). The overall feeling of the advertisement (Y3 and Y4) was found to make an impact on a customer's decision to purchase or not (Dahlén, 2002; Niazi et al., 2012). The conscious decision reported by customers on whether they would make the purchase or not (Y9 and Y10). Padgett and Douglas Allen showed that advertising memorability also plays a role in purchase power (Y11 and Y12). (Padgett and Allen, 1997) Other impacts include: Celebrity endorsement (Y18) (Bocheer and Nanjegowda, 2013; Srikanth et al., 2013); the feature of children or cartoons (Y20 and Y21) (Blatt et al., 1972; Fischer et al., 1991); the language (Y13) (Noriega and Blair, 2008); the narrative style and story-telling (Y16, Y17, Y22) (McQuarrie, 2002; Phillips and McQuarrie, 2002); and sexual-appeal (Y19) is widely established to have an impact on advertisement (Severn et al., 1990; Weller et al., 2015).

**Table 1:** The output dataset list.

	**Output feature**
*Y*_1_(1−7)	Content Quality
*Y*_2_(1−7)	Image Quality
*Y*_3_(1−7)	Excitement
*Y*_4_(1−7)	Attractiveness
*Y*_5_(1−7)	Easiness for understanding
*Y*_6_(1−7)	Clearness of the brand
*Y*_7_(1−7)	Brand awareness
*Y*_8_(1−7)	Familiarness of the brand
*Y*_9_(1−7)	Willingness to buy
*Y*_10_(1−7)	Intention to further learn the product
*Y*_11_(1−7)	likeliness of memorize the advertisement content the next day morning
*Y*_12_(0/1)	If the brand of the product can be memorized the next day
*Y*_13_(0/1)	Chinese language or not
*Y*_14_(0/1)	If it is moving
*Y*_15_(0/1)	If it has significant vision and sound impact
*Y*_16_(0/1)	If it is interesting
*Y*_17_(0/1)	If it surprises you
*Y*_18_(0/1)	Whether it has celebrities
*Y*_19_(0/1)	If it is sexy
*Y*_20_(0/1)	If it has children
*Y*_21_(0/1)	If it has cartoon
*Y*_22_(0/1)	If it is a story telling ads

Eleven of the questions had binary answers of 0 or 1; another eleven were ranked answers from 1 to 7. The questions are listed in each column as output data (see Table 1).

### 2.3. Modeling

Raw EEG data is first augmented into frequency domain EEG signal before creating a larger dataset necessary for analysis. In this paper, the frequency domain EEG signal is listed in Table 2. Further details of input data *X* is listed in section 2.3.1

**Table 2:** The EEG band dataset list.

	**Input feature**	**Meaning**
*X*_1_	Time Stamp	A sequence of numbers indicate the time index of raw signal
*X*_2_	Signal Quality	Value ranges from 0 to 255
*X*_3_	Raw	Voltage
*X*_4_	Attention	Intensity of a user's level of mental focus/attention, value ranges from 0 to 100
*X*_5_	Meditation	Level of a user's mental calmness/relaxation, value ranges from 0 to 100
*X*_6_	Delta	>4 Hz
*X*_7_	Theta	≥4 Hz and <8 Hz
*X*_8_	Low Alpha	7.5–9 Hz
*X*_9_	High Alpha	9.5–12.5 Hz
*X*_10_	Low Beta	12–15 Hz
*X*_11_	High Beta	15–18 Hz
*X*_12_	Low Gamma	30–80 Hz
*X*_13_	High Gamma	>80 Hz

The output data is labeled as *Y*. Each row of output data Y has 22 dimensions, and each dimension represents a feature, and all features are independent. *Y*_450 × 22_ can be written as *Y*_450 × 22_ = [*Y*_1_, *Y*_2_, …, *Y*_22_] where *Y*_*i*_ represents each column vector of Y, and *i* = 1, 2, …, 22, see Table 1.

The purpose of this research is to use SVM to train a machine learning model that can find the map *f*, where *Y* = *f*(*X*), the reason to use Support Vector Machine (SVM) is explained in section 2.3.4. Once found, any new input EEG signals can be translated into a prediction of an advertisement's impact on a consumer, and their likelihood of either a positive impression or willingness to buy the product.

**Figure 1:**
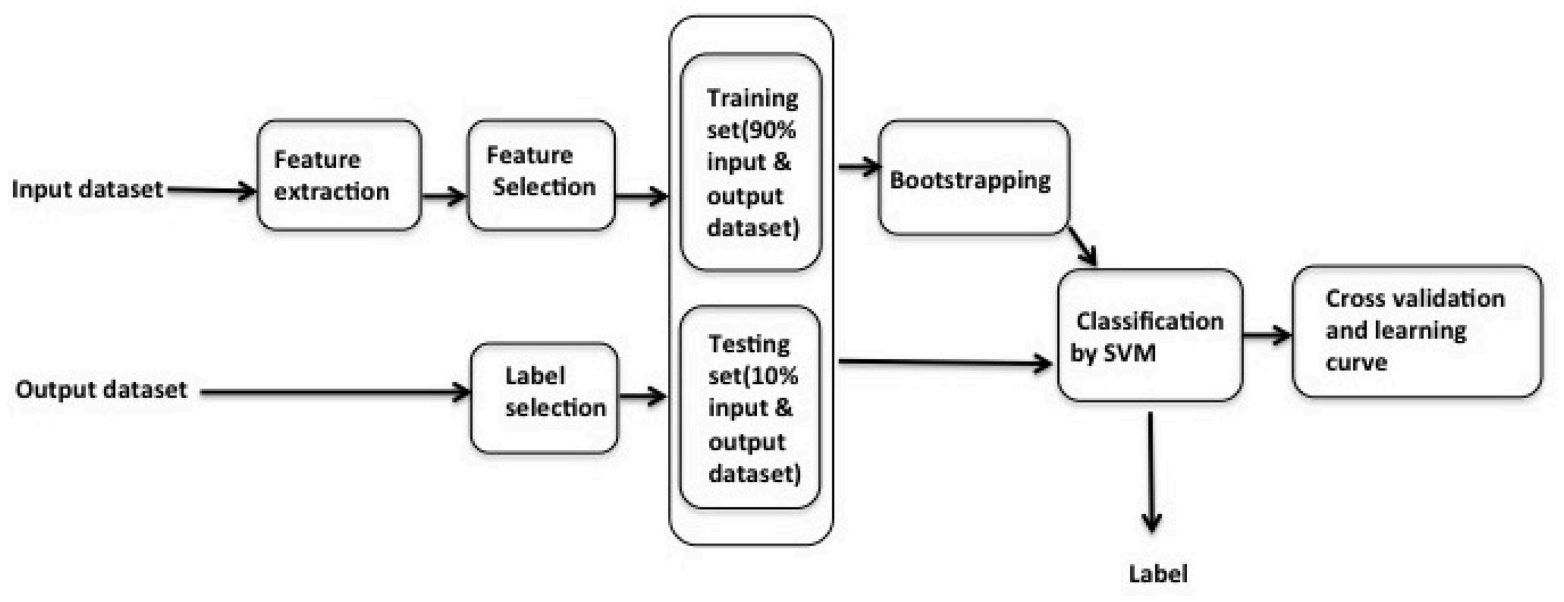
In sample data pre-processing workflow: feature extraction and feature selection are applied in the input dataset to the training dataset, followed by the bootstrapping; label selection is applied in the testing dataset; classification by SVM then applied in the current testing and bootstrapped training dataset, the results of the classification are used to label the data and fed into the cross-validation and learning.

Due to the format of the raw EEG signal data, data extraction is applied to amend the input data into the applicable format. Feature selection is conducted on input data, and label selection is conducted on output data. Among the thirty participants, data corresponding to twenty nine participants are used as in sample data. The sample workflow of this research is shown in Figure 1. 10% of the sample data is then separated as a testing dataset, with the remaining 90% used for training dataset. Bootstrapping is used to bootstrap the training dataset. The data corresponding to the remaining participant is used as out of sample test. The out of sample test is carried out by selecting each last person in the thirty participants and then averaged the thirty out of sample as judgment.

Support Vector Machine is applied as the machine learning method to predict the label given output data. Cross-validation and learning curve is eventually used to check over-fitting/under-fitting. We will elaborate each component in the workflow in the following sections.

#### 2.3.1. Feature extraction

Raw EEG signal is a voltage over time signal. Hence, a crucial process is needed to break the raw EEG signal into constituent frequencies (High alpha/beta/gamma/delta waves), etc. It is crucial because the majority of the informational content in EEG signals are in the frequency domain, so breaking up the signal into constituent frequencies is most appropriate to apply SVM to classify EEG frequency domain data. Besides, according to the research that has been done by Dong et al. (2017), frequency data is better for feature extraction than the raw time domain data. Feature extraction, specifically Fast Fourier transform (FFT) is used to transform the raw EEG signal from time domain into frequencies domain (Heideman et al., 1985; Van Loan, 1992; Pritchard et al., 1994). We computed the EEG frequency band power using traditional EEG frequency band definitions (Delta: 1–3 Hz; theta: 4 Hz, alpha I: 89Hz, alpha II: 1,012 Hz, beta I: 1,317 Hz, beta II: 1,830 Hz, gamma I: 3,140 Hz, gamma II: 4,150 Hz), and these are time signals for each frequency band. Also, because the band EEG signal is collected from the four different product types, and with duration of each advertisement being different, data is also needed to be normalized. Timestamps are used to represent the duration, and advertisement lengths are normalized by cutting advertisements to the same length as the shortest advertisement in its category (it is 24 in cars, 13 in clothing, 28 in digital, and 16 in food).

After normalization and extraction, the raw EEG signal is transformed into a 450 × 13 dimension vector, where each of the elements themselves contain a vector of data corresponding to the advertisement length. The 13 features include wavelength, time, and signal quality and etc. X is a 13 columns, 450 rows vector, *X*_450 × 13_ can be represented by 13 columns, i.e., *X*_450 × 13_ = [*X*_1_, *X*_2_, …*X*_13_], each vector is *X*_*i*_ where *i* = 1, 2…, 12, 13, then each column vector of X is listed in Table 2. However, in the experiment, selected columns X are used in the analysis, and they are Delta, Theta, Low Alpha, HighAlpha, LowBeta, HighBeta, LowGamma and HighGamma, Raw, Mediation and Attention, further selection details are in section 2.3.2.

#### 2.3.2. Feature selection and label selection

The map *Y* = *f*(*X*) can be expressed as *label* = *f*(*features*). It is implied by the equation that a different X and Y will result in different f, and different certainty profiles. Feature extraction and selection is, therefore, a very important process in machine learning

Feature selection can be classified into three types: flat features, stream features, and structured features (Tomasi and Kanade, 1991; John et al., 1994; Chowdhury and Lavelli, 2012). In the experiment, each feature of the input data is independent, hence features in the experiment are classified as flat.

The 13 features are grouped into different combinations to test their ability to predict purchasing chance. In the end, 11 features give the best prediction in the model. These grouped-features also corresponding to the literature that is related to emotion: Delta, Theta, LowAlpha, HighAlpha, LowBeta, HighBeta, LowGamma, and HighGamma (Klimesch, 1999; Teplan, 2002). Attention, Meditation and Raw values (see Table 2 are also considered in the model because their physical meaning is related to emotion. Output data Y is the information of questionnaire answers. In this research, we are using the customer's questionnaire score to determine whether participants will strike an emotion to purchase the product. Each label Y represents different response/measure to the advertisement, if it is 1, it meant positive feedback is linked to emotion. Hence, we label the overall emotion as 1 (positive), and each label in the output data Y is independent. Different people can be influenced by different emotion, so we built a model that considers all emotions equally and then make the prediction. Questionnaire answers can be classified into two types: binary answers (yes or no answers) and ranked answers (values ranked between one and seven). All labels are selected and tested independently to see their significant impact on emotion. Due to the questionnaire answer types, labels are then grouped as binary answers, ranked answers and a combination of binary and ranked answers, each group's signification is also tested to the purchase possibility through emotion.

#### 2.3.3. Data augmentation via bootstrapping

The original dataset contains 450 samples, and is a sparse dataset, and therefore bootstrapping is needed to increase the size of the dataset (Efron and Tibshirani, 1994; Efron et al., 2003). In the experiment, a dataset of twenty-nine people are used as in sample size of 435; while the other participant data of a sample size of 15 is used for out of sample testing. The 435 in sample size is too sparse to divide into a 90% training data (i.e., 393 samples) and a 10% testing data (i.e., 42 samples).

The in-sample data 391 samples are first divided into ten-folds under the condition that each fold has the same ratio of all type of answers in output data Y. Nine of the folds are used for training, while the tenth is used for testing, the ten folds algorithm is shown in Algorithm 2. Bootstrapping is used on each in sample training dataset. In bootstrapping process, Gaussian distribution bootstrapping method has been applied to each column in each training data X, shown in Algorithm 1. Gaussian process is a method to construct a parametric bootstrap approach from Bayesian non-parametric statistics, which implicitly considers the time-dependence of the data. (Efron, 1998) Many features are different frequency bands. Therefore, Gaussian is suitable also because it can generate continuous new data. However, Gaussian is a probability distribution between 0 and 1, while the data can be numbers even bigger than 1,000 and any number between 0 and 1 is too small to be the new bootstrapped number to be used in the experiment, hence in the bootstrapping process, a scalar has been calculated to multiply the probability, and the scalar is calculated as *scale* = 0.5 × (*min* + *max*), where the min represents the minimum number of that column, and the max represents the maximum number of that column. The new bootstrapped number is the original value plus the scaled Gaussian probability. In this way, the original data spreading will not be affected. Mathematical combination is then applied in the ninefold training data, which is shown in Algorithm 3. The corresponding output data Y is duplicated to match the size of bootstrapped training data X in each of the bootstrapping processes.

**Algorithm 1:** Bootstrapping1

1:	**procedure** GAUSSIAN PROCESS BOOTSTRAP TO EACH COLUMN
2:	**Input :** Gaussian model, input data **Xtrain**
3:	**Output :** edited input data **X_p_**
4:	⊳ Use Gaussian process regression bootstrap the whole **Xtrain** dataset, name as bootstrapping1
5:	**for** Each column in **Xtrain** as *i* **do**
6:	⊳ Edit **Xtrain** as **X_p_**
7:	**X_p_** = **Xtrain**
8:	⊳ select the maximum element
9:	*max* = *max*(**X**_**p***i*_)
10:	⊳ select the minimum element
11:	*min* = *min*(**X**_**p***i*_)
12:	*scale* = 0.5 × (*min* + *max*)
13:	⊳ bootstrapping using Gaussian Distribution in (−1, 1)
14:	*bootc* = *scale* × *Gaussian*_*bootstrap*(**X_p_**) + **X_p_**
15:	⊳ update **X_p_** with all new columns
16:	**X_p_** = *bootc*

**Algorithm 2:** Tenfolds

1:	**procedure** TEN-FOLDS: IN SAMPLE TRAINING AND TESTING DATA SELECTION THROUGH INDEXES
2:	⊳ Ten-fold the whole dataset into ten sub dataset: same ratio of ones and zeros in nine subsets, combine the rest of unselected zeros and ones into the tenth subset
3:	**Input :** input data **X**, output data **Y**
4:	**Output :** nine different training input/output dataset **Xtrain_i_**
5:	⊳ Find the index of ones in the **Y**
6:	*index*_*ones* = *index*_*of*_*ones*(**Y**)
7:	⊳ Find the index of zeros in the **Y**
8:	*index*_*zeroes* = *index*_*of*_*zeroes*(**Y**)
9:	⊳ Calculate the ratio of ones in the length of **Y**
10:	*ratio* = *length*(*index*_*ones*)/*length*(**Y**)
11:	⊳ After divide the **Y** into ten folds, find the number of ones in each fold
12:	*ones* = *round*(*ratio* * *length*(**Y**)/10)
13:	⊳ After divide the **Y** into ten folds, find the number of zeros in each fold
14:	*zeros* = *round*((1 − *ratio*) * *length*/10)
15:	⊳ Find the left over ones after first nine folds
16:	*ones*_*left* = *length*(*index*_*ones*) − 9 * *ones*
17:	⊳ Find the left over zeros after first nine folds
18:	*zeros*_*left* = *length*(*index*_*zeros*) − 9 * *zeros*
19:	⊳ In the tenth fold, append the selected index of zeros and ones
20:	**Xfold**[10] = *append*(**X**[*ones*_*left*], **X**[*zeros*_*left*])
21:	**Yfold**[10] = *append*(**Y**[*ones*_*left*], **Y**[*zeros*_*left*])
22:	for *i* in 0:9 **do**
23:	⊳ Find the **Y** index of ones in each fold
24:	*selection*_*ones* = *index*_*of*_*ones*[i*ones :(*i*+1) * *ones*]
25:	⊳ Find the **Y** index of zero in each fold
26:	*selection*_zeros = *index*_*of*_*zeros*[*i* * zeros :(*i* + 1) * *zeros*]
27:	⊳ Find the **Y** index in each fold
28:	*selection* = *append*(*selection*_*ones*, *selection*_*zeros*)
29:	⊳ Find the **Yfold** that is corresponding to the selected column index in **Y**
30:	**Yfold**[*i*] = **Y**[*selection*]
31:	⊳ Find the **Xfold** that is corresponding to the selected column index in each **X**
32:	**Xfold**[*i*] = **X**[*selection*]
33:	⊳ **Xtrain** and **Ytrain** is achieved by appending any nine of ten subsets in Xfold and Yfold
34:	**Xtrain** = *append*(**Xfold**[*C*(10, 9)])
35:	**Ytrain** = *append*(**Yfold**[*C*(10, 9)])

**Algorithm 3:** Bootstrapping2

1:	**procedure** BOOTSTRAPPING THE TRAINING X DATASET FROM TENFOLDS USING MATHEMATICAL COMBINATION
2:	**Input :** input data **Xtrain**
3:	Output : nine different training input dataset **X2_j_**
4:	⊳ Start with the original dataset **Xtrain**, but edit the selected column by the corresponding column in algorithm1
5:	**X2** = **Xtrain**
6:	**for** Each column in **X2** as *j* **do**
7:	⊳ make a copy of **X2**_*j*_ for editing
8:	*column* = **X2_j_**
9:	**for** *r* in *range*(1, 12) **do**
10:	⊳ generate all unique iterations of *column* using *nCr* method, and then replace the corresponding index in the **Xtrain** while the rest of the columns remain the same
11:	*newdata* = *nCriterations*(12, *r*, *row*)
12:	**X2_j_** = *replace*_*bootstrapping1*(**Xtrain**[*j*]_*replace* **Xp**[*newdata*])

#### 2.3.4. Support vector machine

In literature, there are many different classification selection processes in machine learning. For example, Navie Bayes classifier, Random Forest, K-Nearest Neighbors, and Decision Tree (Quinlan, 1987; Altman, 1992; Ho, 1995; Rennie et al., 2003). Support vector machine is a machine learning model used for classification and regression analysis (Aizerman et al., 1964; Boser et al., 1992; Jin and Wang, 2012). When SVM is used for classification, they separate a given set of binary labeled training data and a hyperplane that is maximally distance from them. Assume the input data is ${\text{x}}^{\text{j}}=(x_{1}^{j}...x_{n}^{j})$ be the realization of the random vector **x**^**j**^ While ϕ is the map mapping the feature space to a label space **y**, where label space contains many vectors, mathematically label as {(**x**^1^, *y*^1^), …(**x**^*m*^, *y*^*m*^)}. The SVM learning algorithm finds a hyperplane (**w**, *b*) such that the quantity

(1) $$\gamma =min_{t}y^{i}\{<w,\phi (x^{i})>-b\}$$

is maximized. In this equation, the dimension of ϕ is the same as the dimension of the label y and < **w**, *f*(**x**^*i*^) > −*b* corresponds to the distance between point **x**^*i*^ and the decision boundary. γ is the margin and b is a real number. The kernel of this function is $K_{i,j}=<\phi \left(x^{i}\right),\phi \left(x^{j}\right)>$. Given a new data *x* to classify, a label is assigned according to its relationship to the decision boundary, and the corresponding decision function is written as *f*(*x*) = *sign*(< *w*, ϕ(*x*) > −*b*).

In the experiment, as each label in the output is independent, and are binary (ranked answers are binarized via threshold as described below), therefore, regression that can end up with binary results are tested, i.e. non-linear regression, logistic regression and Support Vector Regression. All three methods have been utilized and proven appropriate in the previous research in EEG emotion detection (Schröder et al., 2001; Wang et al., 2011; Bejaei et al., 2015). However, Support Vector Machine is more appropriate on this type of sparse data (Wang et al., 2011). Besides, the experiment SVM has been shown to give the highest prediction accuracy result. The number of samples is larger than the number of features in our experiment, therefore the variable is chosen to reflect so; the loss function is set to 'Squared_hinge' because it is commonly used in classification and 'Squared_hinge' is convex and smooth and matches the function 0 − 1, which is suitable for our experiment. 'Squared_hinge' is mathematically written as (1 − *yf*(*x*))^2^. The map f is linear. Therefore the kernel is zero. Regularization is *L*2 as output data is no longer sparse, nor feature selection as features are targeted in output data (Ng, 2004).

In order to combine both binary and ranked answers, the rank answers are binarized using a thresholds according to the following rules:

- When the selected labels involve only the ranked answers, the threshold is set to be 3.5, 4 (i.e. = (7 + 1)/2) and 4.5.
- When the selected labels involve only binary answers, the threshold is set to be 0, 0.5 (i.e. = (0 + 1)/2 and 1.
- When the Threshold is a combination both types of answers, then the threshold can be written as equation: *Thre* = α ^*^ *Thre*_7_ + β ^*^ *Thre*_2_, where α and β are the weights

#### 2.3.5. Cross-validation and learning curve

When the SVM model is built up, cross-validation and learning curve is used to assess whether the model is over-fitted or under-fitted (Geisser, 1993; Kohavi et al., 1995; Babyak, 2004; Frost, 2015).

## 3. Result

### 3.1. Behavior result

In this model, we use labels and EEG signals as variables, and emotion is the hidden variable bridge labels and EEG signals, they can be written as *Y* = *f*(*X*|*E*), where E is emotion. The result of this experiment shows that if we have an individual's EEG signal, which has been collected from the single electron device after watching the advertising, the accuracy of predicting if this individual would or not make a purchase of the corresponding product in the advertising is around 75% based on the SVM model. In the experiment, each participant has been selected as the out sample data, and then the rest of the participants are in sample data (90% of in sample data is the training dataset, while the 10% of the in-sample data is the testing dataset, bootstrapping has been applied to the 90% in sample training dataset.

The accuracy of prediction using SVM over the ranked answers is 77.28%. In this setting, the threshold of ranked answers results is 4. The recall score of this model is 72% and the F score for this model is 75%. In the same threshold of ranked answers result is 4, each category prediction is: the likelihood of purchasing the car is 63.5%, the likelihood of purchasing the cloth is 92.3%, the likelihood of purchasing the digital is 68.5% and the likelihood of purchasing the food is 82.76% (Table 3).

**Table 3:** Accuracy prediction of the ranked answer to different type of product at different thresholds.

**Product type**	**Threshold 3.5**	**Threshold 4**	**Threshold 4.5**
Car	0.365384615385	0.634615384615	0.826923076923
Food	0.603448275862	0.827586206897	0.931034482759
Digital	0.388888888889	0.685185185185	0.87037037037
Clothes	0.673076923077	0.923076923077	0.961538461538
All dataset	0.595090082962	0.772837217714	0.898908153808

The accuracy of prediction using SVM model over combined ranked and binary answers is 75.4% under the conditions that ranked and binary answers have equal weighting (of 0.5), the threshold of ranked answers is 4, the threshold of a binary answer is 0.4, the threshold of the whole dataset is 0.4 (Table 8). The recall score of this model is 69% and the F score for this model is 71%. In the same experiment setting, each category prediction is: the likelihood of purchasing the car is 59.6%, the likelihood of purchasing the clothes is 92.3%, the likelihood of purchasing the digital is 64.8% and the likelihood of purchasing the food is 81.03% (Tables 4–7).

**Table 4:** Likelihood of purchasing the car in the combined answer model at different thresholds.

**Threshold to each type answer in car**	**Threshold of the car dataset**		
	***Thre* = 0.4**	***Thre* = 0.5**	***Thre* = 0.6**
*Thres*_7_ = 0.4, *Thres*_2_ = 3.5	0.365384615385	0.365384615385	0.826923076923
*Thres*_7_ = 0.4, *Thres*_2_ = 4	0.596153846154	0.596153846154	0.865384615385
*Thres*_7_ = 0.4, *Thres*_2_ = 4.5	0.75	0.75	0.903846153846
*Thres*_7_ = 0.5, *Thres*_2_ = 3.5	0.365384615385	0.365384615385	0.903846153846
*Thres*_7_ = 0.5, *Thres*_2_ = 4	0.634615384615	0.634615384615	0.903846153846
*Thres*_7_ = 0.5, *Thres*_2_ = 4.5	0.807692307692	0.807692307692	0.923076923077
*Thres*_7_ = 0.6, *Thres*_2_ = 3.5	0.365384615385	0.365384615385	0.980769230769
*Thres*_7_ = 0.6, *Thres*_2_ = 4	0.634615384615	0.634615384615	0.980769230769
*Thres*_7_ = 0.6, *Thres*_2_ = 4.5	0.807692307692	0.807692307692	1

**Table 5:** Likelihood of purchasing the food in the combined answer model at different thresholds.

**Threshold to each type answer in food**	**Threshold of the food dataset**		
	***Thre* = 0.4**	***Thre* = 0.5**	***Thre* = 0.6**
*Thres*_7_ = 0.4, *Thres*_2_ = 3.5	0.603448275862	0.603448275862	0.965517241379
*Thres*_7_ = 0.4, *Thres*_2_ = 4	0.810344827586	0.793103448276	0.965517241379
*Thres*_7_ = 0.4, *Thres*_2_ = 4.5	0.913793103448	0.913793103448	0.98275862069
*Thres*_7_ = 0.5, *Thres*_2_ = 3.5	0.603448275862	0.603448275862	0.98275862069
*Thres*_7_ = 0.5, *Thres*_2_ = 4	0.827586206897	0.827586206897	0.98275862069
*Thres*_7_ = 0.5, *Thres*_2_ = 4.5	0.931034482759	0.931034482759	1
*Thres*_7_ = 0.6, *Thres*_2_ = 3.5	0.603448275862	0.603448275862	0.948275862069
*Thres*_7_ = 0.6, *Thres*_2_ = 4	0.827586206897	0.827586206897	0.948275862069
*Thres*_7_ = 0.6, *Thres*_2_ = 4.5	0.931034482759	0.931034482759	0.948275862069

**Table 6:** Likelihood of purchasing the digital in the combined answer model at different thresholds.

**Threshold to each type answer in digital**	**Threshold of the digital dataset**		
	***Thre* = 0.4**	***Thre* = 0.5**	***Thre* = 0.6**
*Thres*_7_ = 0.4, *Thres*_2_ = 3.5	0.407407407407	0.407407407407	0.944444444444
*Thres*_7_ = 0.4, *Thres*_2_ = 4	0.648148148148	0.648148148148	0.962962962963
*Thres*_7_ = 0.4, *Thres*_2_ = 4.5	0.814814814815	0.814814814815	0.981481481481
*Thres*_7_ = 0.5, *Thres*_2_ = 3.5	0.388888888889	0.388888888889	0.981481481481
*Thres*_7_ = 0.5, *Thres*_2_ = 4	0.685185185185	0.685185185185	0.981481481481
*Thres*_7_ = 0.5, *Thres*_2_ = 4.5	0.87037037037	0.87037037037	0.981481481481
*Thres*_7_ = 0.6, *Thres*_2_ = 3.5	0.388888888889	0.388888888889	0.981481481481
*Thres*_7_ = 0.6, *Thres*_2_ = 4	0.685185185185	0.685185185185	0.981481481481
*Thres*_7_ = 0.6, *Thres*_2_ = 4.5	0.87037037037	0.87037037037	NA

**Table 7:** Likelihood of purchasing the clothes in the combined answer model at different thresholds.

**Threshold to each type answer in clothes**	**Threshold of the clothes dataset**		
	***Thre* = 0.4**	***Thre* = 0.5**	***Thre* = 0.6**
*Thres*_7_ = 0.4, *Thres*_2_ = 3.5	0.653846153846	0.653846153846	1
*Thres*_7_ = 0.4, *Thres*_2_ = 4	0.923076923077	0.923076923077	1
*Thres*_7_ = 0.4, *Thres*_2_ = 4.5	0.961538461538	0.961538461538	1
*Thres*_7_ = 0.5, *Thres*_2_ = 3.5	0.692307692308	0.692307692308	1
*Thres*_7_ = 0.5, *Thres*_2_ = 4	0.923076923077	0.923076923077	1
*Thres*_7_ = 0.5, *Thres*_2_ = 4.5	0.961538461538	0.961538461538	1
*Thres*_7_ = 0.6, *Thres*_2_ = 3.5	0.692307692308	0.692307692308	0.980769230769
*Thres*_7_ = 0.6, *Thres*_2_ = 4	0.923076923077	0.923076923077	0.980769230769
*Thres*_7_ = 0.6, *Thres*_2_ = 4.5	0.961538461538	0.961538461538	0.980769230769

**Table 8:** Likelihood of purchasing in the combined answer model at different thresholds.

**Threshold to each type answer in whole dataset**	**Threshold of the whole dataset**		
	***Thre* = 0.4**	***Thre* = 0.5**	***Thre* = 0.6**
*Thres*_7_ = 0.4, *Thres*_2_ = 3.5	0.6044	0.60022153401	0.940276239347
*Thres*_7_ = 0.4, *Thres*_2_ = 4	0.754142459932	0.758595745643	0.950177453263
*Thres*_7_ = 0.4, *Thres*_2_ = 4.5	0.866265795601	0.866265795601	0.968578339399
*Thres*_7_ = 0.5, *Thres*_2_ = 3.5	0.595090082962	0.595090082962	0.977326672243
*Thres*_7_ = 0.5, *Thres*_2_ = 4	0.772837217714	0.772837217714	0.977326672243
*Thres*_7_ = 0.5, *Thres*_2_ = 4.5	0.894454868097	0.894454868097	0.977326672243
*Thres*_7_ = 0.6, *Thres*_2_ = 3.5	0.595090082962	0.595090082962	0.977326672243
*Thres*_7_ = 0.6, *Thres*_2_ = 4	0.772837217714	0.772837217714	0.977326672243
*Thres*_7_ = 0.6, *Thres*_2_ = 4.5	0.894454868097	0.894454868097	0.977326672243

This indicates that EEG collected using single-electrode wearable devices is above 70% accuracy for prediction whether customers would purchase the product after watching the advertisement, and it can achieve higher accuracy prediction and reach about 75%.

The out of sample predictions are tested in two cases, and in each case, the results are the average/mean of picking up different thirty individual participants as out sample test. When it is only ranked answers, if the samples from the remaining participant from all four categories are combined into one dataset, then the prediction of purchasing power can reach 72.4%; if each product category is tested, then the car purchasing power can reach up to 50.09%, the clothing purchasing power can reach 78.01%, the digital products purchase power can reach 56.56% and the food purchase power can reach 69.34%. When it is the combined result of ranked and binary answers, if the samples from the remaining participant from all four categories are combined into one dataset, then the prediction of purchasing power can reach 71.2%; if each product category is tested, then the car purchasing power can reach up to 51.8%, the clothing purchasing power can reach 65.9%, the digital products purchase power can reach 57.2% and the food purchase power can reach 60.62%.

Also, in the research, each label Y has been tested, but it is a difficult test and the result is not balanced, the recall and F scores are both low, that is why those predictions are not mentioned in the results. Different thresholds for each model have also been tested during the experiment, however, they are not good enough to accurately explain the model.

Seventy-five percentage is a relatively high result to judge the purchasing power based on the EEG signal after watching the advertising, though there is no research on the direct link of EEG signal and purchase intention. However, research has been done on using emotions to quantify the purchasing power. In 2006, John Pawle and Peter Cooper showed that emotional factors to brand decision making range from 63 to 85% (Tsai, 2005; Pawle and Cooper, 2006).

### 3.2. Cross-validation and learning curve

The two models with the whole dataset are selected to draw the cross-validation and learning curve. The reasons for only selecting these two are they give the best prediction results, and they are directly related to the paper hypothesis: predicting the likelihood of customers purchasing the products.

**Figure 2:**
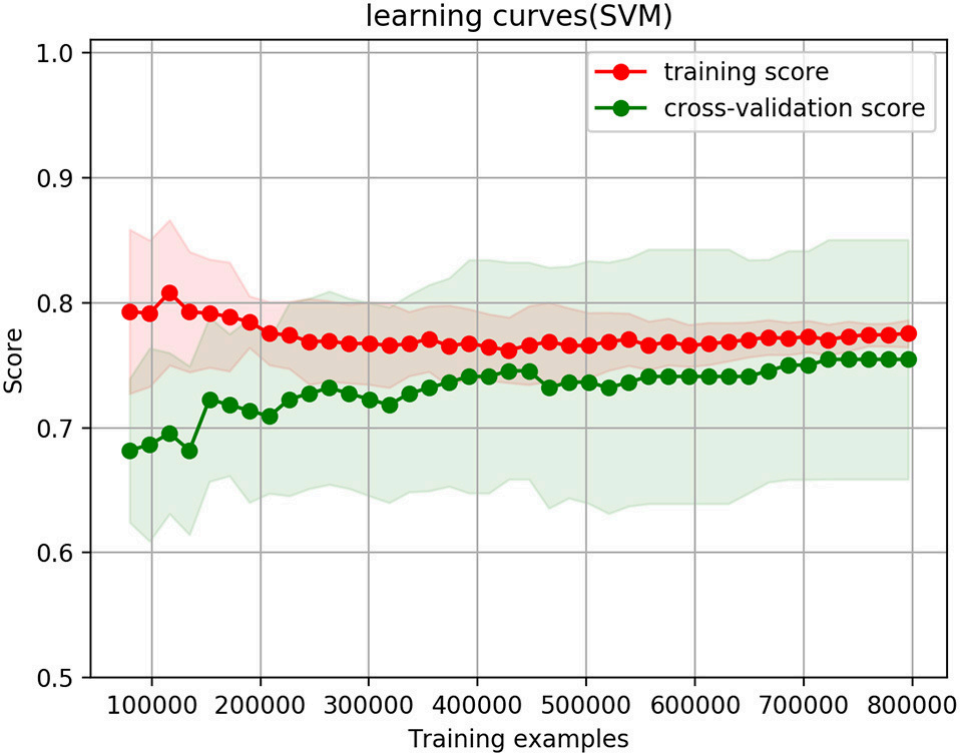
Cross validation curves when only ranked answers are selected, with a threshold of 4.

**Figure 3:**
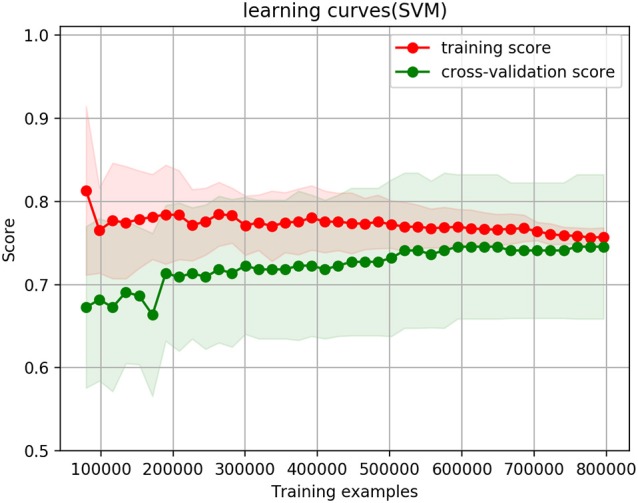
Cross-validation curves when thresholds of ranked answers is 4 and binary answers threshold is 0.4, their weight is equal at 0.5, and the whole dataset thresholds is 0.4.

The cross-validation and learning curve is shown in Figures 2, 3.

Figure 2 shows the cross-validation and learning curve when only the ranked answers are selected. Figure 3 shows the cross-validation and learning curve when the thresholds of ranked answers is 4, and binary answers threshold is 0.4, their weight is equal at 0.5, and the whole dataset thresholds is 0.4. In both figures, the X-axis represents the size of training examples; Y-axis represents the accuracy prediction score. The red lines represent the training score, and green lines represent cross-validation score/testing score.

In the ranked answers only case in Figure 2, training and testing curves converge to 77.28%; when the combination of ranked answers and binary answers are selected (ranked answers threshold is 4, and binary answers threshold is 0.4), as shown in Figure 3, the training and testing curves converge to 75.4%. The shaded red and shaded green are standard deviations at confidence interval 10% to its corresponding training score and corresponding testing score. Mathematically, they are calculated as (*training*_*mean*_ − *training*_*std*_, *training*_*mean*_ + *training*_*std*_) and (*testing*_*mean*_ − *testing*_*std*_, *testing*_*mean*_ + *testing*_*std*_). Both figures show that the training curves are slightly over-fitting at the beginning, but it gradually decrease and reach a stable point with the decreasing variance. In testing curves, the variance remains approximately the same from the beginning till the end throughout the training example size increases; it remains the same because the testing data size does not change regardless of how training example size changes. Convergence of training and testing curves means there is no over-fitting and no under-fitting issues of the model; the SVM model we set up and use therefore performs well.

## 4. Discussion

The approximately 75% accuracy prediction and the converging cross-validation curve result together show that the EEG signal and edited AIDA metrics model we found is a suitable model. This result is sufficient to declare EEG a useful dataset to collect in the advertising industry.

Further improvements can be made to the accuracy of prediction through improving the chosen thresholds and increasing the study sample size rather than relying on bootstrapping. The accuracy of prediction may be further improved through selecting both male and female participants and gender-neutral products and their advertisement.

The statistical results show that the ranked answers with a threshold of 4 has a better result than the combined rank and binary answers, with about 2% more accuracy. It also shows that each category prediction is lower than the whole dataset prediction in both models. It shows products like cars have the lowest accuracy of prediction and clothes have the highest accuracy of prediction among the four categories. This may be caused by cars being a costly product, and the decision to purchase one depends more on lifestyle and personal circumstance than advertising. In this case, the prediction accuracy can be improved by pre-selecting participants to control for these factors, or by adjusting predictions based on the personal circumstances. In the experiment, the F scores and the recall scores are also good in the model; it happens to be consistent that the whole dataset scores are better than each category dataset. The out of sample test result is not as good as the sample test, but it is acceptable, which further indicates the model is good.

In the current research, the emotions and opinions of the user have been used as a hidden variable to bridge EEG signal and self-reported metrics to evaluate the impact of advertisement and its power to influence purchasing. In the literature, research has been done on the relationship of Theta wavelength with emotion and the relationship of Beta Wavelength with emotion (Lucchiari and Pravettoni, 2012; Wang et al., 2016). Considering that EEG signals contain components at many other frequencies, it is worth further investigation of the relationship between EEG signal and emotion, and how they impact a decision to make a purchase.

In past attempts, an AIDA model has been used to quantify emotion metrics in terms of scores for attention, interest, desire and action aspects after watching the advertisement (Strong, 1925). In our research, we follow the idea of the AIDA model and add additional dimensions to quantify these emotions to achieve a more precise measure. The ranked and binary answers have equal waiting in accordance with the AIDA model and literature mentioned in section 2.1 which considers all labels with equal impact. It is also interesting to further execute a non-parametric machine learning model to assess the importance of each label or a grouping of similar labels for purchasing power. More details on the emotion model our experiment uses are described in section 2.1.

This is a new method in Neuromarketing. The advantage of this method is to be able to assess impact fast. The model, once found, can work in real-time on EEG signals, in a highly automated way. Something not possible in traditional methods that involve data collection via surveys or discussion, and then analysis, which requires both time and man-power to accomplish; or assessing the impact through sales figures afterwards, which can be easier to automate, but has much longer cycle times.

Our proposed evaluation method also shows less bias due to EEG data being involuntary and therefore not subject to conscious and experimental bias. Unlike in a focus group study conducted by an advertisement company, the study in an academic setting removes the potential for study biases in which participants tend to give kinder answers than they may think, out of goodwill or some sense of social obligation (De Pelsmacker et al., 2002; Maison et al., 2004; Shen and Li, 2009). The questionnaire questions are written neutrally, and asks similar questions from slightly different angles to validate consistency. Because these questions yield consistent results, it indicates a high likelihood that they represent the true thought of participants. The questions in the questionnaire do not include identifiable or confidential information of any individual; therefore removing potential reasons for participants to hide their true opinion.

Furthermore, the device used in this research is a single-electrode wearable device that is low cost, portable, and easy to use, allowing this kind of data collection to be scaled much more rapidly than machines traditionally associated with functional brain imaging in neuroscience, such as traditional clinical-grade multi-channel EEG setups, and fMRI (Signal, 2015). Multiple types of EEG devices have been studied in the literature and applied to neuromarketing studies; the results do not show an appreciable improvement in accuracy of multi-channel EEG systems vs. single electrode EEG systems (Hamzy and Dutta, 2000; Liu et al., 2013). Therefore, single electrode EEG devices is sufficient for the experiment, and the ease of use of a simple, self-contained and battery-operated wearable device opens up new kind of customer engagement opportunities where their EEG can be recorded throughout the day in settings that are much more normal than would be the case in a lab setting, removing any influence that may come from the experimental setup.

The most significant part of this research is the extension of modeling emotions with a single-wavelength collected from EEG to multidimensional wavelengths, allowing the extraction of more informational content from EEG than previously attempted.

The result of this research can be further applied to individual consumers behavior; to allow the advertising industry to tailor their advertisement for maximum impact; and adapted to work for TV an movie studios to predict viewership rates of movies and TVs from trailers.

## 5. Conclusion

We proposed a model that can use EEG signals measured using low-cost consumer-grade EEG headsets taken while a consumer watches an advertisement, to rapidly predict the consumer's likelihood of purchasing the product. While further research can be made on the selection of thresholds, and the quality of the result can be improved with the collection of larger datasets, the method as shown is nevertheless easy to deploy, yields rapid results, scales better than any existing method, and introduces less experimental and environmental bias. If employed in place of existing focus-group studies, any company involved in mass-media advertising stands to improve the effectiveness of their advertising, improve estimates of the impact on sales of their advertising, and be more informed when building their advertising strategy, leading to increased ROI.

## Ethics statement

The experiment was carried out at ZheJiang University and the approval was obtained from the Ethics committee at the ZheJiang University, China. In addition, written informed consent forms were obtained from all volunteers before the experiment started. The ethics approval number from ZheJiang University is NSL20160012.

## Author contributions

ZW carried out the experiment and wrote the paper. CW and AS edited the paper. PW prepared the experiment. XW prepared the experiment data and provided advice with some of the editing. YG supervised the project.

### Conflict of interest statement

The authors declare that the research was conducted in the absence of any commercial or financial relationships that could be construed as a potential conflict of interest.
